# Impact of Short-Term Air Pollution on Respiratory Infections: A Time-Series Analysis of COVID-19 Cases in California during the 2020 Wildfire Season

**DOI:** 10.3390/ijerph19095057

**Published:** 2022-04-21

**Authors:** Lilian Ouja Ademu, Jingjing Gao, Onah Peter Thompson, Lawrence Anebi Ademu

**Affiliations:** 1 Public Policy Ph.D. Program, College of Liberal Arts and Sciences Charlotte, University of North Carolina at Charlotte, Charlotte, NC 28223, USA; jgao9@uncc.edu (J.G.); pthomp34@uncc.edu (O.P.T.); 2 Department of Animal Production and Health, Federal University Wukari, Wukari 1020, Nigeria; ademu@fuwukari.edu.ng

**Keywords:** air pollution, respiratory infections, COVID-19, environmental health, Generalized additive models, Feasible Generalized Least Squares Model, California, wildfires

## Abstract

The 2020 California wildfire season coincided with the peak of the COVID-19 pandemic affecting many counties in California, with impacts on air quality. We quantitatively analyzed the short-term effect of air pollution on COVID-19 transmission using county-level data collected during the 2020 wildfire season. Using time-series methodology, we assessed the relationship between short-term exposure to particulate matter (PM_2.5_), carbon monoxide (CO), nitrogen dioxide (NO_2_), and Air Quality Index (AQI) on confirmed cases of COVID-19 across 20 counties impacted by wildfires. Our findings indicate that PM_2.5_, CO, and AQI are positively associated with confirmed COVID-19 cases. This suggests that increased air pollution could worsen the situation of a health crisis such as the COVID-19 pandemic. Health policymakers should make tailored policies to cope with situations that may increase the level of air pollution, especially during a wildfire season.

## 1. Introduction

The World Health Organization formally announced a global pandemic from a new coronavirus in March 2020 [1]. The illness which is caused by the virus called SARS-CoV-2 produces respiratory symptoms that are fatal in some cases. Current evidence suggests that the virus is spread through close contact with other humans, primarily through coughing or sneezing [2]. The coronavirus disease (COVID-19) continues to spread, with emerging variants of the virus. Millions of cases and deaths have been reported globally. In the United States alone, millions of cases have been recorded, with thousands of deaths resulting from complications related to the disease [3].

To alleviate the damages caused by the COVID-19 pandemic, numerous studies have examined the possible factors contributing to the transmission of the disease. Pioneer studies focused on person-to-person transmission and analyzed how factors such as population mobility play a significant role in virus transmission [4,5]. Other studies explored how contaminated environmental surfaces aid the transmission of the virus from an infected to an uninfected person [6,7]. Research on the impact of short-term exposure to pollutants such as particulate matter (PM), carbon monoxide (CO), sulfur dioxide (SO_2_), nitrogen dioxide (NO_2_), and ozone (O_3_) on the transmission of COVID-19 is still emerging, and comparatively scarce.

Before the COVID-19 pandemic, past studies have linked pollutants to adverse health conditions [8,9]. Nitrogen dioxide (NO_2_), for example, has been linked to chronic obstructive pulmonary disease and asthma [10]. Other studies suggest that particulate matter such as PM_10_ and PM_2.5_ could act as a platform for respiratory virus transmission [11,12,13,14]. Air quality and environmental conditions have also been associated with lung infections caused by viruses [15]. There are also indications that air pollution weakens the immune system and may increase one’s chances of contracting respiratory viral infections such as COVID-19 [16,17]. A recent study suggests that a 1 μg/m^3^ increase in PM_2.5_ is associated with an 8% increase in the COVID-19 death rate [18]. 

Though air quality varies with weather conditions, some cities experience poor air quality for extended periods due to environmental disasters such as volcanic eruptions and wildfires. This paper examines the relationship between short-term exposures to pollutants such as PM_2.5_, NO_2_, CO, and overall air quality (AQI) and confirmed cases of COVID-19 during the 2020 wildfire season in California. We model the effect of these pollutants using time series methods. We apply Generalized Additive Models (GAMs) for estimating the relationship while controlling for meteorological factors, day-fixed effects, and county fixed effects. In one of the sensitivity analyses, we use a Feasible Generalized Least Squares Model (FGLS) model to check the robustness of our findings. The study focuses on 20 counties impacted by the wildfires between February and December 2020.

### 1.1. The 2020 California Wildfires and COVID-19

The peak of the COVID-19 pandemic in the United States in 2020 coincided with the most significant wildfire season recorded in modern California’s history [19,20]. The 2020 California wildfire season began in February and lasted for months with the major fire outbreaks occurring in the months from August to December [21]. By the end of the fire season in December, the fires had destroyed 4,257,863 acres of land [21,22]. Though particulate matter, oxides of nitrogen, and sulfur pollution declined sharply in many parts of the world during the pandemic, wildfire days in California recorded higher amounts of PM_2.5_ pollution [23,24,25]. This highlights the need to investigate if higher daily concentrations of pollutants are associated with increased COVID-19 cases in counties that experienced wildfires in California.

Past research has linked wildfire smokes to adverse health outcomes [26,27,28]. The Environmental Protection Agency describes wildfire smoke as a public health concern comprising a mix of gaseous pollutants such as carbon monoxide (CO) and particulate matter. Studies indicate that particulate matter, which contains solid and liquid suspension, poses a significant risk to public health during a wildfire [29]. Furthermore, elevated CO levels outdoors during a fire can be of particular concern for those with some preexisting conditions such as heart disease. This paper analyzed data from 20 counties impacted by the wildfires. We use 1 February 2020, to 31 December as our study period. This was selected for two reasons; COVID-19 data is available from February 2020, and the 2020 California fire season lasted until December 2020.

### 1.2. Long-Term and Short-Term Air Pollution and COVID-19

Both long and short-term exposure to air pollutants may be a complex factor in increasing SARS-CoV-2 transmission and lethality [30]. A study in 71 provinces across Italy suggests that chronic air quality was highly correlated with COVID-19 cases suggesting that chronic exposure to air pollution may predispose people to the disease [2]. Similarly, another study found that long-term exposure to high amounts of PM_2.5_ is associated with increased mortality from COVID-19 [18]. A study in Spain examined the spatial spread of COVID-19 using a mixed longitudinal ecological design. Their results suggest that chronic exposure to NO_2_ and PM_10_ are predictors of the spatial spread of the virus [31]. Another study employed an ecological analysis to examine the relationship between chronic exposure to pollutants and reported cases of COVID-19 in Canada. They applied a negative binomial regression model and found positive associations between long-term exposure to PM_2.5_ and COVID-19 incidence [32]. 

There is still relatively less research on the impact of short-term exposure to pollutants on COVID-19 transmission. Recent studies report mixed findings and use different methodologies. A study using Generalized additive models (GAM) found positive associations between moving average concentrations of pollutants such as NO_2_, PM_2.5_, O_3_, and COVID-19 cases in China [33]. Another examined the spatial relationship between PM_10_ and PM_2.5_ and COVID-19 deaths. Their results suggest a positive relationship between pollutants and COVID-19 deaths [34]. However, another research found negative relationships between COVID-19 and these pollutants including NO_2_ and SO_2_ in California [35]. This study, however, employed fundamental techniques such as Spearman and Kendall correlation for their statistical analysis. 

Using data from Los Angeles and Ventura counties in the US, another study applied a generalized linear model (GLM) to examine the same relationship [36]. They also found negative relationships between daily SARS-CoV-2 cases and pollutants PM_2.5_ and PM_10_. Though they applied a dynamic emission model to further strengthen their analysis, the study focused only on two counties, and they did not control for the daily SARS-CoV-2 test which is highly correlated to the number of confirmed cases recorded across counties. A more recent study found positive associations between exposure to PM_2.5_ in the short-term and COVID-19 cases and deaths using data collected during the 2020 wildfire season in 92 western U.S. counties [25]. However, this study did not examine other air pollutants like CO and NO_2_. They also did not adjust for the confounding effects of SARS-CoV-2 tests in their models. 

Since the daily levels of pollutants are higher on wildfire days than on non-wild fire days [25], the 2020 California wildfire season provides an opportunity to estimate the relationship between short-term exposure to pollutants and confirmed cases of COVID-19. This paper extends the current literature on the impacts of short-term pollution on health by exploiting the wildfire season in California and applying different time series methodologies to further investigate this relationship. We first use Generalized additive models in the main analyses to analyze the short-term impact of pollutants such as PM_2.5_, CO, NO_2,_ and overall air quality index (AQI) on confirmed COVID-19 cases. We then use a Feasible Generalized Least Squares Model in one of the four sensitivity analyses to check the robustness of our findings.

## 2. Materials and Methods

### 2.1. Study Area and Period

**Figure 1:**
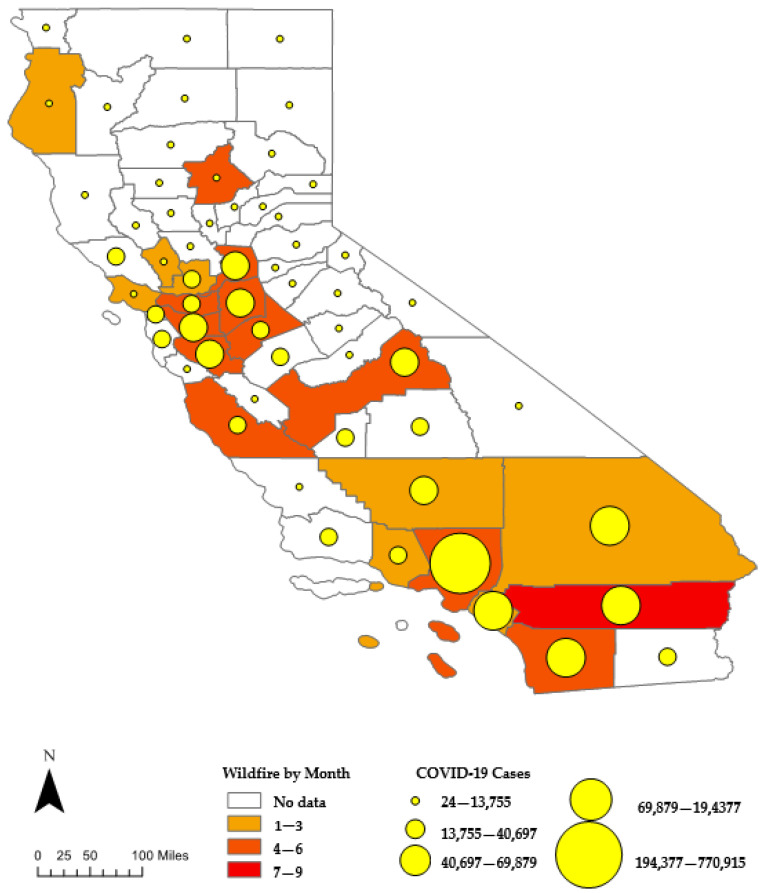
Locations of the 20 counties and cumulative COVID-19 cases in each as of 31 December 2020.

In 2020, California was one of the states most impacted by COVID-19 in the USA [35]. The number of COVID-19 cases and deaths resulting from COVID-19 in the state of California in 2020 was over 1.9 m and 26,000 respectively [37]. As of February 2022, California is the state with the highest confirmed cases of the disease in the United States [38]. This study includes 20 out of the 53 counties affected by the 2020 California wildfires (See Figure 1 and Table A1 in the Appendix A). 

We focus on these counties due to the incompleteness of meteorological data on other counties. Furthermore, only counties where fires burned more than 1000 acres and led to significant structural damage or casualties were selected for this study. The study focused on the period 1 February 2020, to 31 December for two reasons; COVID-19 data is available from February 2020, and the 2020 California fire season which started with the first fire outbreak in February, lasted until December 2020 (See Table A1 in Appendix A).

### 2.2. Data Collection

#### 2.2.1. COVID-19 Data

COVID-19 data in the state of California is made available to the public by the California Department of public health [39]. This data includes total SARS-CoV-2 tests and confirmed COVID-19 cases. This study uses data collated from 1 February 2020, to 31 December 2020, for each of the 20 counties. 

#### 2.2.2. Air Pollution Data 

Data on air pollution during the study period was obtained from open-source data available on the United States Environmental Protection Agency (EPA) website [40]. Data on pollutants such as PM_2.5_, carbon monoxide (CO), and nitrogen dioxide (NO_2_) were collected for the 20 counties included in the study. To estimate the impact of the overall air quality on the number of COVID-19 cases, data on the air quality index (AQI) across the twenty counties were also collated from the EPA website for statistical analysis. The EPA uses a standard formula for its AQI computation. Reported data on AQI uses the highest value calculated among the pollutants used for estimating the index [41]. This study uses the reported values as a continuous variable in the statistical analysis.

#### 2.2.3. Meteorological Data

Data on daily mean temperature, mean precipitation, and average wind speed during the study period was obtained from the National Centers for Environmental Information, an agency under the National Oceanic and Atmospheric Administration [42]. 

### 2.3. Statistical Analyses

Time-series methods have been established as very useful tools for examining the impact of air pollution and meteorological factors on health outcomes over time [25,33,43,44]. Previous studies demonstrate that the impact of air pollutants can linger for several days [33,44] so a moving average approach was applied in all our analyses to capture the lagged effect of each pollutant examined in the study [25,33,45]. Our choice for moving averages (lag 0–7, lag 0–14, or lag 0–21) is based on the official COVID-19 incubation period issued by the US Centers for Disease Control and Prevention [46]. We used multiples of seven since research on the incubation period of the virus is still very dynamic and ranges from 7 days to 21 [33,47]. 

#### 2.3.1. Generalized Additive Models (GAMs)

A Generalized Additive Model with a gaussian distribution family, was used to estimate the relationship between the moving average concentrations (lag 0–7, lag 0–14, and lag 0–21) of all the pollutants and daily confirmed cases of COVID-19 [33,48]. To avoid multicollinearity, we used separate models to analyze the effect of the air pollutants and air quality (AQI) on COVID-19 cases across the twenty counties [49]. The model used for each analysis is described below:

log(*cases_it_*) = *a* + *X_ig_* + s(*temp_it_*) + s(*prec_it_*) + s(*wind_it_*) + log(*tests_it_*) + *cases_it_*_-1_ + *county_i_* + *day_t_* + *ε_it_* (1)

Here, *a* is the intercept. log(*cases_it_*) captures the number of COVID-19 cases on day *t* in county *i.* 1 was added to each original count to ensure that the logarithm of 0 is not captured [25,33,48]. *X_ig_* is the linear term of a specific moving average concentration (where *g* = lag 0–7, lag 0–14, or lag 0–21) of air pollutants and AQI in county *i* [36,50]. Meteorological factors: daily mean temperature (*temp_it_*), precipitation (*prec_it_*), and wind speed (*wind_it_*) on day *t* in county *i* were included in the model to control for possible confounding effects from meteorological factors. *cases_it-_*_1_ was introduced to control for potential autocorrelation from previous cases of COVID-19. In addition, county fixed (*countyi*) effects and day fixed effects (*day_t_*) were included to account for the fixed effects specific to each county and daily effects such as lockdowns affecting all counties respectively. s(.) captures the smooth function (k = 9 with thin plate spline function with a maximum of 3 degrees of freedom) of each nonlinear term in the model. Lastly, the log of the daily number of SARS-CoV-2 tests (*tests_it_*) reported in every county was controlled for since the number of confirmed COVID-19 cases is highly correlated with the number of SARS-CoV-2 tests in every county. 1 was added to each original number of tests to ensure that the logarithm of 0 is not captured. All analyses were done using the “mgcv” package in R statistical software (version 3.5.2).

#### 2.3.2. Sensitivity Analysis

We carried out four sensitivity analyses to check the robustness of the results. The first three sensitivity analyses were done using the GAM. First, Los Angeles (the most populated county and the county with the highest number of confirmed cases) was excluded from the data to check if the findings would remain robust afterward. Los Angeles county makes up almost 38% of the confirmed COVID-19 cases in the data analyzed. 

Secondly, we used moving average lagged terms for all the meteorological variables in the model. Like pollutants, meteorological conditions can linger for days and may have a lagged impact on health outcomes [51,52,53]. Past research recommends using lag 9 to lag 13 for environmental analysis [52]. We used lag 9 in this sensitivity analysis. In the third sensitivity analysis, the lagged term (*cases_it-_*_1_) for confirmed cases of COVID-19 was excluded from each of the models since we were analyzing daily COVID cases and not cumulative COVID-19 cases.

Finally, we used an FGLS model to account for intragroup heteroskedasticity and serial correlation, The “pggls” function in the R allows the error covariance structure inside every group of observations (the counties in this study) to be unrestricted and therefore robust against any type of intragroup heteroskedasticity and serial correlation [54]. Unlike the GAM above, we did not include day-fixed effects in this analysis. We accounted for only the residual spatial effect using *county_i_* as shown in the model below. We used separate models to analyze the effect of air pollutants and air quality (AQI) on COVID-19 cases across the twenty counties. The model used for each analysis is described below:

log(*cases_it_*) = *a* + *X_ig_* + *temp_it_* + *prec_it_* + *wind_it_* + *tests_it_* + *cases_it_*_-1_ + *county_i_* + *ε_it_* (2)

The description of all variables in the above model remains the same as described in equation 1 above. All the analyses were done using the “plm” package in R statistical software (version 3.5.2).

## 3. Results

### 3.1. Descriptive Analysis

**Figure A1:**
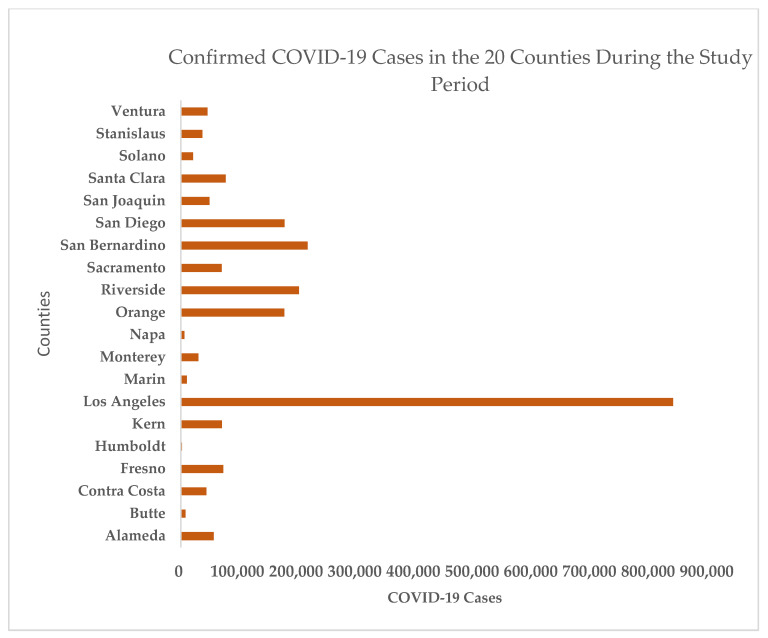
Confirmed COVID-19 cases in the 20 counties during the study period.

Table 1 shows the descriptive statistics for air pollution variables, meteorological variables, and daily confirmed COVID-19 cases for the study period, February to December 2020 across the twenty counties. We split the data into the peak months when most of the fires occurred (August to December) and the off-peak months (February to July). The months when fires occurred in each of the 20 counties are shown in Table A1. Compared to the off-peak months, the values on the measures of pollution during the peak months were relatively higher. The average daily concentration of PM_2.5_, CO, NO_2,_ and AQI was 21.33 μg/m^3^, 0.43 ppm, 10.14 ppb, and 59.78 respectively during peak months. During the off-peak months, the average daily concentration of PM_2.5_, CO, NO_2,_ and AQI was 7.48 μg/m^3^, 0.23 ppm, 5.52 ppb, and 29.83 respectively. The average daily number of COVID-19 cases during the peak period and off-peak months was 553 and 142 respectively. We also show the overall COVID-19 numbers for all the counties in the study in Figure A1.

### 3.2. Time Series of Confirmed COVID-19 Cases and Pollutants in the 20 Counties in Our Analysis

**Figure 2:**
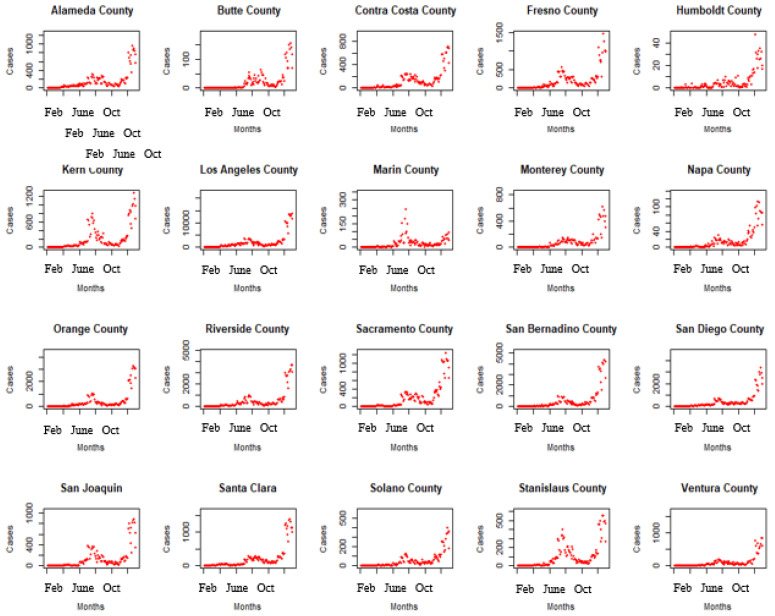
Time series of confirmed COVID-19 cases in the 20 counties during the study period.

**Figure 3:**
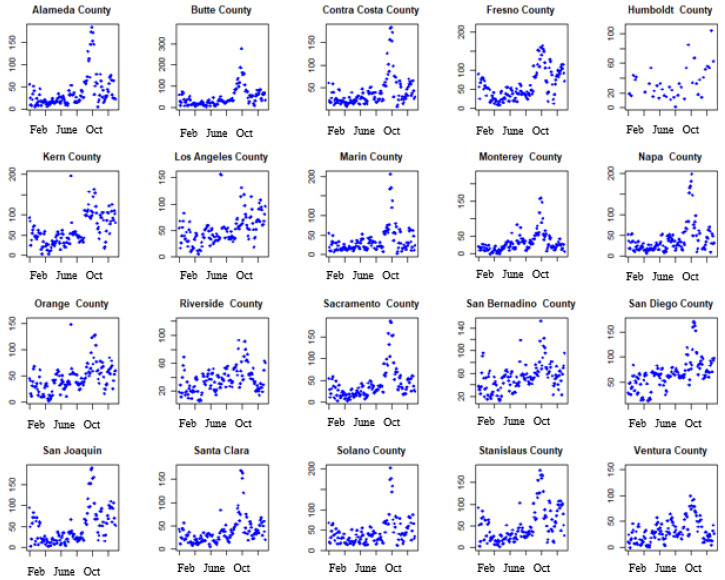
Time series of air quality (AQI) in the 20 counties during the study period.

Figure 2 shows the time series trends of the daily number of confirmed COVID-19 cases for the twenty counties during the study period. There appears to be an overall increasing trend in the number of confirmed cases of COVID-19 across all the counties during the study period. Similarly, Figure 3 shows the time series trend of the overall air quality (AQI) for all the counties during the study period. There appears to be an overall increasing trend in air pollution during the study period with sharp peaks occurring between August and December across all the counties in the study.

### 3.3. Relationship between Air Pollution and Confirmed Cases of COVID-19

Table 2 shows the results of the GAM models. Shown are the coefficients highlighting the relationship between the explanatory variables (air pollutants and AQI) and confirmed COVID-19 cases. All the coefficients show statistical significance at conventional levels. We observed positively significant associations between PM_2.5_, CO, AQI, and confirmed COVID-19 cases across all the lags. For a lag 0–7 moving average, a 1 μg/m^3^ increase in PM_2.5_ is associated with a 0.4% increase in the daily counts of COVID-19. A 1 unit increase in CO is associated with a 36% increase in the daily counts of COVID-19. A one-unit increase in AQI is associated with a 0.3% increase in the daily counts of COVID-19. Lastly, NO_2_ was negatively associated with confirmed COVID-19 cases; a 1 unit increase in NO_2_ was associated with a 0.7% decrease in the daily counts of COVID-19.

#### Sensitivity Analysis

Table A2, Table A3 and Table A4 in the Appendix A show the results of the three sensitivity analyses done using the GAM. Generally, the results indicate that the relationships between confirmed COVID-19 cases and pollutants are robust across all three models. In Table A2 (after Los Angeles was excluded from the data set used for the analysis), the statistical significance and effects sizes are still very close to those in Table 2 above. Similarly, Table A3 and Table A4 further confirm the robustness of our findings. Even after using a moving average of lag 9 on all the meteorological factors and excluding the lagged term (*cases_it_*_-1_), the statistical significance and effects sizes remain almost identical. Table A5 in the Appendix A shows the results of the sensitivity analysis with the FGLS model. Like the other models, the statistical significance of the coefficients is very similar even though the effect sizes are larger. The only important change is in the direction of effect for NO_2_ from a negative to a positive effect. All other pollutants maintain their directions of effect.

## 4. Discussion

In this paper, we employed time series methods to explore the relationship between air pollution and daily confirmed COVID-19 cases. We observed significant associations for all the pollutants examined in this study. As demonstrated in previous studies, the effect of air pollution can linger for several days after incidents [33,44]. Our choice for moving averages (lag 0–7, lag 0–14, or lag 0–21) is based on previous studies and official statements on the COVID-19 incubation period issued by the US Centers for Disease Control and Prevention [46,47]. Our findings show that PM_2.5_, CO, and AQI are all significantly and positively associated with confirmed COVID-19 cases in all the moving average lags. However, the GAM results show a negative relationship between NO_2_ and COVID-19 cases. These results remained robust in all the sensitivity analyses with the GAM. In the sensitivity analyses using the FGLS model, we find positive associations between all the pollutants and confirmed COVID-19 cases. These findings suggest that air pollution could play an important role in COVID-19 transmission.

Many studies have shown that air pollution is correlated to respiratory infections caused by microorganisms [55,56]. Air quality and environmental conditions have been associated with lung infections caused by viruses [15]. Some studies suggest that particulate matter such as PM_10_ and PM_2.5_ could act as a platform for virus transmission [11,12,13,14]. There are also indications that air pollution weakens the immune system and may increase one’s chances of contracting respiratory viral infections such as COVID-19 [16,17]. We made comparisons between the findings in this study and previous studies to check for similarities and differences. In one study [57], short-term exposure to higher PM_2.5_ was correlated with higher confirmed cases of acute lower respiratory infection using an observational cross-over design. Another study that combined a generalized Poisson regression model and a distributed lag nonlinear model (DLNM) found significant associations between atmospheric particulate matter (PM_2.5_ and PM_10_,) and hospitalizations for respiratory diseases in China [58]. 

Other studies specific to COVID-19 have also found significant associations between air pollution and the disease. Using distributed lag models, a study found that increased exposure to PM_2.5_ was associated with increased COVID-19 cases and deaths across 92 counties in the US [25]. Another study using a GAM found significant relationships between confirmed cases of COVID-19 and six air pollutants in China [33]. As in our study, these two studies consistently found positive associations between pollutants (PM_2.5_, CO, and NO_2_) and confirmed COVID-19 cases. Unlike our study, other studies done in the US have found negative associations between confirmed cases of COVID-19 and the pollutants analyzed in our study [35,36,51]. However, the methodologies employed in these studies were very different from the ones used in our study. 

Some studies suggest that exposure to certain concentrations of NO_2_ could reduce susceptibility to respiratory viral infections [59,60]. This could be the reason for the negative relationship observed in the results in the GAM models used in this study. Additional research is needed to understand the biological mechanisms behind this phenomenon observed not just in our study but in others [35,36,51].

This study has several limitations. For one, it focused on associations and not causal effects of air pollution and indicators of air pollution on confirmed cases of COVID-19. For example, more people could have stayed indoors during the wildfires which may have increased their chances of contracting the virus. Studies indicate that close spaces with poor air circulation and inhalation of aerosols from infected persons increase the spread of respiratory viruses [61,62]. 

This study also focused on 20 out of the 53 counties impacted by the 2020 California wildfires. While this was in part due to the incompleteness of data on some pollution and meteorological indicators, a complete sample of the counties affected would have improved the validity of our findings. We also did not account for economic or social factors that could have increased COVID-19 risk. Many studies indicate that certain socioeconomic groups have been disproportionately impacted by COVID-19 [63]. Lastly, this study did not consider sub-group analysis in terms of demographics such as gender, occupation, or race. Future studies should check for possible heterogeneity across socio-demographic groups.

## 5. Conclusions

This study suggests that there is a significant relationship between air pollution and confirmed cases of COVID-19. Short-term exposure to increased concentrations of PM_2.5_, CO, and higher values of AQI is associated with an increased risk of COVID-19. These results remained robust in all the sensitivity analyses done in this study, Our findings also suggest that short-term exposure to a higher concentration of NO_2_ is related to decreased risk of COVID-19 infection. This finding calls for further research to understand this phenomenon.

This study has obvious importance for the management of COVID-19 transmission or future pandemics of respiratory diseases. For a more nuanced public health advisory, policymakers should pay close attention to regions with more predisposition to forest fires and inadvertently, higher measures of air pollution. This is due to the fact that these regions may be disproportionately impacted by respiratory diseases such as COVID-19.

## Figures and Tables

**Table 1:** Descriptive statistics of measures of air pollution, meteorological variables, and daily confirmed new cases across all counties during the peak and off-peak months of the 2020 California wildfire season.

	The Peak Months of Fire Outbreaks (August–December 2020)			Off-Peak Months (February–July)		
	Mean (SD)	Min	Max	Mean (SD)	Min	Max
PM_2.5_ (μg/m^3^)	21.33 (13.00)	0.30	329	7.48 (5.49)	0	142
CO (ppm)	0.43 (0.24)	0.09	2.70	0.23 (0.12)	0	1.09
NO_2_ (ppb)	10.14 (7.08)	0.25	49.79	5.52 (4.79)	0	33.17
Mean temperature °F	64.71 (13.24)	31.00	110	61.01 (11.27)	30.86	89.78
Precipitation (mm)	0.02 (0.09)	0	1.46	0.04 (0.14)	0	1.6
Average Wind speed (m/s)	5.31 (2.55)	0.67	21.03	7.09 (2.76)	0.67	26.62
Air Quality Index (AQI)	59.78 (38.40)	1	379	29.83 (17.18)	0	196
Daily confirmed cases	553 (1573)	0	22,264	142 (359)	0	3613

**Table 2:** Results of the GAM models.

Pollutants/Air Quality	Lag (0–7) Days	Lag (0–14) Days	Lag (0–21) Days
	Cases	Cases	Cases
PM_2.5_	0.004 ***	0.004 ***	0.004 ***
	(0.001)	(0.001)	(0.001)
CO	0.363 ***	0..355 ***	0.347 ***
	(0.075)	(0.087)	(0.093)
NO_2_	−0.007 **	−0.009 **	−0.008 **
	(0.003)	(0.003)	(0.003)
AQI	0.003 ***	0.003 ***	0.002 ***
	(0.0005)	(0.0005)	(0.0006)

Significance codes: ‘***’: 0.001, ‘**’: 0.01.

## Data Availability

The data that supports the findings of this study are available on request from the corresponding author (L.O.A).

## Appendix A

**Table A1:** Counties included in the study and the months with fire incidents in each (Source: fire.ca.gov accessed on 24 January 2022 ).

County	Date of First Fire Incident in the County	Containment Date of Last Fire Incident in the County	Months of Fire
Alameda	4-July	1-October	July, August, September, October
Butte	8-May	22-October	May, June, July, August, October
Contra Costa	27-April	12-October	April, May, June, July, October
Fresno	12-June	9-November	June, July, August, September, October, November
Humboldt	15-February	24-October	February, July, October
Kern	17-June	12-August	June, July, August
Los Angeles	1-July	7-November	July, August, September, October, November
Marin	18-August	2-October	August, September, October
Monterey	7-June	31-December	June, August, September, October, December
Napa	22-May	25-October	May, September, October
Sacramento	12-June	4-October	June, July, August, September, October
Orange	26-October	10-December	October, November, December
Riverside	3-March	14-December	March, May, June, July, August, September, October, December
San Bernardino	3-July	16-November	July, October, November
San Diego	9-June	3-December	June, July, August, September, December
San Joaquin	17-June	1-October	June, July, August, September, October
Santa Clara	4-June	1-October	June, July, August, September, October
Solano	3-June	9-July	June, July
Stanislaus	8-June	1-October	June, August, September, October
Ventura	10-June	8-December	June, August, December

**Table A2:** GAM models after excluding Los Angeles County.

Pollutants/Air Quality	Lag (0–7) Days	Lag (0–14) Days	Lag (0–21) Days
	Cases	Cases	Cases
PM_2.5_	0.004 ***	0.004 ***	0.004 ***
	(0.0019)	(0.001)	(0.001)
CO	0.367 ***	0.379 ***	0.393 ***
	(0.073)	(0.094)	(0.095)
NO_2_	−0.008 ***	−0.0076 *	−0.005
	(0.003)	(0.003)	(0.003)
AQI	0.003 ***	0.003 ***	0.002 ***
	(0.0005)	(0.0005)	(0.0005)

Signif. codes: ‘***’: 0.001, ‘*’: 0.05.

**Table A3:** GAMs using a moving average of lag 9 on all the meteorological factors.

Pollutants/Air Quality	Lag (0–7) Days	Lag (0–14) Days	Lag (0–21) Days
	Cases	Cases	Cases
PM_2.5_	0.005 ***	0.005 **	0.004 ***
	(0.001)	(0.001)	(0.001)
CO	0.499 ***	0.485 ***	0.450 ***
	(0.076)	(0.089)	(0.094)
NO_2_	−0.005 ^†^	−0.006 *	−0.006 ^†^
	(0.003)	(0.003)	(0.003)
AQI	0.003 ***	0.003 ***	0.003 ***
	(0.0005)	(0.0005)	(0.0006)

Signif. codes: ‘***’: 0.001, ‘**’: 0.01, ‘*’: 0.05, ‘^†^’: 0.1.

**Table A4:** GAM after excluding (*cases_it-_*_1_) of previous cases of COVID-19 from the model.

Pollutants/Air Quality	Lag (0–7) Days	Lag (0–14) Days	Lag (0–21) Days
	Cases	Cases	Cases
PM_2.5_	0.004 ***	0.005 **	0.004 ***
	(0.001)	(0.001)	(0.001)
CO	0.305 ***	0.283 **	0.285 **
	(0.074)	(0.086)	(0.091)
NO_2_	−0.008 **	−0.009 **	−0.009 **
	(0.003)	(0.003)	(0.003)
AQI	0.002 ***	0.003 **	0.003 *
	(0.0005)	(0.0005)	(0.0005)

Signif. codes: ‘***’: 0.001, ‘**’: 0.01, ‘*’: 0.05.

**Table A5:** Result of the FGLS model.

Pollutants/Air Quality	Lag (0–7) Days	Lag (0–14) Days	Lag (0–21) Days
	Cases	Cases	Cases
PM_2.5_	0.012 ***	0.017 ***	0.015 *
	(0.001)	(0.001)	(0.006)
CO	1.585 ***	2.220 ***	3.244 ***
	(0.125)	(0.232)	(0.492)
NO_2_	0.036 ***	0.064 ***	0.070 ***
	(0.001)	(0.0005)	(0.011)
AQI	0.010 ***	0.011 ***	0.012 ***
	(0.001)	(0.001)	(0.003)

Signif. codes: ‘***’: 0.001, ‘*’: 0.05.
